# The role of environmental factors on transmission rates of the COVID-19 outbreak: an initial assessment in two spatial scales

**DOI:** 10.1038/s41598-020-74089-7

**Published:** 2020-10-12

**Authors:** Canelle Poirier, Wei Luo, Maimuna S. Majumder, Dianbo Liu, Kenneth D. Mandl, Todd A. Mooring, Mauricio Santillana

**Affiliations:** 1 Computational Health Informatics Program, Boston Children’s Hospital, Boston, MA 02215 USA; 2 Department of Pediatrics, Harvard Medical School, Boston, MA 02215 USA; 3 Department of Biomedical Informatics, Harvard Medical School, Boston, MA 02215 USA; 4 Department of Earth and Planetary Sciences, Harvard University, Cambridge, MA 02138 USA; 5 Harvard T.H. Chan School of Public Health, Boston, MA 02215 USA

**Keywords:** Statistical methods, Infectious diseases, Diseases

## Abstract

First identified in Wuhan, China, in December 2019, a novel coronavirus (SARS-CoV-2) has affected over 16,800,000 people worldwide as of July 29, 2020 and was declared a pandemic by the World Health Organization on March 11, 2020. Influenza studies have shown that influenza viruses survive longer on surfaces or in droplets in cold and dry air, thus increasing the likelihood of subsequent transmission. A similar hypothesis has been postulated for the transmission of COVID-19, the disease caused by SARS-CoV-2. It is important to propose methodologies to understand the effects of environmental factors on this ongoing outbreak to support decision-making pertaining to disease control. Here, we examine the spatial variability of the basic reproductive numbers of COVID-19 across provinces and cities in China and show that environmental variables alone cannot explain this variability. Our findings suggest that changes in weather (i.e., increase of temperature and humidity as spring and summer months arrive in the Northern Hemisphere) will not necessarily lead to declines in case counts without the implementation of drastic public health interventions.

## Introduction

Since December 2019, an increasing number of pneumonia cases caused by a novel coronavirus (SARS-CoV-2) have been identified in Wuhan, China^1,2^. This new pathogen has exhibited high human-to-human transmissibility with approximately 16,819,944 confirmed cases of COVID-19 and 662,000 deaths reported globally as of July 29, 2020.

On January 23, 2020, Wuhan—a city in China with 11 million residents—was forced to shut down both outbound and inbound traffic in an effort to contain the COVID-19 outbreak ahead of the Lunar New Year. However, it is estimated that more than five million people had already left the city before the lockdown^3^, which has led to the rapid spread of COVID-19 within and beyond Wuhan.

In addition to population mobility and human-to-human contact, environmental factors such as absolute humidity (defined as the water content in ambient air) and temperature, have been found to be strong environmental determinants of transmissions for some viral pathogens^4,5^. For example, influenza viruses survive longer on surfaces or in droplets in cold and dry air, thus increasing the likelihood of subsequent transmission. For COVID-19, a recent study found that higher temperatures may have led to higher transmission in 122 cities in China, concluding that there was no evidence supporting the hypothesis that case counts of COVID-19 would decline when temperatures increase^6^. In contrast, another study showed that higher transmission was observed in colder places when analyzing data from 429 cities across the world, suggesting that temperature could potentially impact COVID-19 transmission^7^. A third study found that warm and dry weather was favorable to the survival of the virus^8^ whereas a fourth determined that transmission would decrease with the arrival of spring and summer^9^. As discussed in a recent paper^10^, quantifying the relationship between COVID-19 transmission and weather variables is a challenging task for multiple reasons. First, characterizing the time evolution of COVID-19 transmission from the available datasets produced by multiple public health agencies can yield very different temporal outbreak trajectories. Second, estimating the instantaneous transmission rate, Rt, using the dates of report as opposed to using the dates of onset of symptoms will invariably lead to significantly different results. Third, the choice of methods to calculate R_t_ using for example Cori’s method or Wallinga and Teunis’ method, will lead to temporal shifts that complicate establishing causal relationships between weather and transmission^11^. Fourth, non-pharmaceutical interventions to contain COVID-19 in China since January 23, 2020 significantly reduced the country-wide disease duration and outdoor transmission^12^; the environmental impact on transmission may have been eclipsed as a consequence. Finally, differences in reporting practices across regions may complicate any efforts to compare relationships between weather and transmission from one location to another.

Despite these challenges and inconsistent conclusions from research on this topic to date, it is important to propose alternative methodologies that provide a complementary understanding of the effects of environmental factors on the ongoing outbreak to support decision-making pertaining to disease control. This is especially true for locations where the risk of transmission may have been underestimated, such as humid and warm places.

### Our contribution

Here, we propose a methodology that can be implemented in real-time during the early phase of an outbreak to examine variability in environmental factors, mobility, and transmission of COVID-19 across provinces and cities in China. We show that the observed spatial patterns of COVID-19 transmission are not explained by ambient temperature, absolute humidity or human mobility alone. Our findings do not support the hypothesis that high absolute humidity in warmer environments may limit the survival and transmission of this new virus.

## Data and methods

### Epidemiological data

To conduct our analysis, we collected epidemiological data from the Johns Hopkins Center for Systems Science and Engineering website^13^. Incidence data were collected from various sources, including the World Health Organization (WHO); U.S. Centers for Disease Control and Prevention (CDC); China CDC; European CDC; the Chinese National Health Center (NHC); as well as DXY, a Chinese website that aggregates NHC and local China CDC situation reports in near real-time. Daily cumulative confirmed incidence data were collected for each province in China from January 22, 2020 to February 26, 2020. We also obtained epidemiological data for other affected countries, including Iran, Italy, Singapore, Japan, and South Korea and 345 cities in China.

### Estimation of a proxy for the reproductive number

Based on the cumulative incidence data for each province, city or country, we estimated a proxy for the reproductive number *R* in a collection of 5-, 6- and 7-day intervals^14^. *R* is a measure of potential disease transmissibility defined as the average number of people a case infects before it recovers or dies. Our proxy for *R*, designated as *R*_*proxy*_, is a constant that maps cases occurring from time (*t*) to time (*t* + *d*) onto cases reported from time (*t* + *d*) to time (*t* + *2d*); where *d* is an approximation of the serial interval (i.e., the number of days between successive cases in a chain of disease transmission). For multiple time points, *t*, we obtained values of *R*_*proxy*_*(t,d)*, given by:

$${R}_{proxy}\left(t,d\right)=\frac{C\left(t+2d\right)-C\left(t+d\right)}{C\left(t+d\right)-C\left(t\right)}$$

where *C* is the cumulative case count up to time *t*, and the values of *d* range from [5 to 7]. Our measure is considered only a proxy for *R* because it does not use details of the (currently imprecise definition of the) serial interval distribution, but instead, simply calculates the multiplicative increase in the number of incident cases over approximately one serial interval. Such proxies are at least approximately monotonically related to the true reproductive number and cross 1 when the true reproductive number crosses 1^15^, i.e. increases in our proxy typically signal increases in R. After computing these proxy values over a variety of subsequent moving time windows, for each serial interval (5, 6 and 7 days), a mean value was obtained and used as our estimated reproductive number *R* for each province, city, and country.

### Time windows

Our study was conducted from January 22, 2020 to February 26, 2020 to make sure that there was COVID-19 activity across all the locations. Indeed, the main outbreaks in Chinese provinces took place from the beginning of January to the end of February. In addition, to characterize the temporal evolution of the COVID-19 outbreak (a large decrease in transmission after the closure of Wuhan and a subsequent flattening of the epidemic curve), the reproductive number *R*_*proxy*_ was calculated for two different time periods. The first one, τ_1_, was from January 22, 2020 to February 8, 2020 and the second one, τ_2_, was from February 9, 2020 to February 26, 2020. In our study, the reproductive numbers computed on the first and second time periods are labeled R0_τ1_ and R0_τ2_, respectively.

### Weather data

All meteorological data for this study were taken from the ERA5 reanalysis, a state-of-the-art data product produced at the European Centre for Medium-Range Weather Forecasts^16,17^. ERA5 is generated by using a vast range of meteorological observations to constrain a physics-based numerical weather prediction model. This procedure, referred to by atmospheric scientists as data assimilation, yields a globally complete gridded data set including many different meteorological variables. Time resolution of ERA5 is quite high (1 h) and it is also frequently updated (preliminary ERA5 data are available 5 days behind real time), making it useful for studies of rapidly evolving disease outbreaks^18^. Furthermore, a conceptually similar but much less sophisticated data product (the National Centers for Environmental Prediction-National Center for Atmospheric Research reanalysis^19^) has been found useful for studies of influenza epidemics^5^.

We obtained relevant ERA5 data at a spatial resolution of 0.25° (~ 28 km at the equator). We represented weather conditions in each city of interest by those in the ERA5 grid box containing the city. Because we assumed that the majority of disease incidence for each province occurs in or near the capital due to increased population density in these areas, we chose to represent each province’s weather conditions by those in the ERA5 grid box containing the provincial capital. Near-surface air temperature, used in this study, is one of the standard ERA5 variables. Absolute humidity (more specifically, near-surface water vapor density) is not one of the standard ERA5 output variables. Instead, it must be computed from variables that are available, namely near-surface air temperature (*T*_*2*_) and near-surface dew point temperature (*T*_*d*_) (see supplementary material for more details). We produced hourly time series of temperature and humidity and then computed time mean absolute humidities and temperatures over January 17–31, 2020 and February 1–15, 2020, for comparison to τ1 and τ2 *R*_*proxy*_ data, respectively.

### Human mobility data

We obtained mobility data made publicly available by the Chinese Internet search engine Baidu^20^. From the full origin–destination matrix for each day, we created a dataset to get the percentage of people traveling from Wuhan and going to the different Chinese provinces from January 1, 2020 to January 22, 2020 (i.e., before the mandated lockdown in Wuhan.)

### Data analysis

Given the potential noise contained in the reported case counts, we tested the robustness of our findings by gradually removing provinces and cities for which their data was deemed too noisy or missing from our analysis. This was done in three subsequent filtering steps as follows. First, we included all provinces and cities where *R*_*proxy*_ could be properly calculated (i.e. enough cases were reported). Second, we removed provinces where mobility data was not available. Finally, we removed provinces and cities where the values of *R*_*proxy*_ were unrealistically high (due perhaps to reporting biases), specifically above 3. The latter filter was used to further remove potential noisy values that would affect our analysis and responding to the fact that the World Health Organization has estimated that R values range from 2 to 2.5. For country-level transmission, we did not conduct any statistical analysis due to the extremely noisy values of *R*_*proxy.*_

### Human mobility as a predictor of the reproductive number

To disentangle if our reproductive number estimates could be explained by importation of cases from Wuhan, Hubei, alone; and if they could be interpreted as indicators of local transmission, we formulated a linear model with the local *R*_*proxy*_ as the response variable, and human mobility as a predictor at the province level. Specifically, we used mobility data before the closure of Wuhan (i.e. from January 1, 2020 to January 22, 2020) to explain $\mathrm{R}{0}_{\uptau_1}$.

$$\mathrm{R}{0}_{\uptau_1}\left(j\right)={\upbeta }_{0}+{\upbeta }_{1}{X}_{mobility}\left(j\right)+\upepsilon \left(j\right)$$

where $\mathrm{R}{0}_{\uptau_1}$(j) is the proxy for the reproductive number for the province *j* during the immediate time-period of two weeks after Wuhan's lockdown; and ${\mathrm{X}}_{\mathrm{mobility}}$ is the percentage of people traveling from Wuhan and $\upepsilon$ $\sim \mathcal{N}\left(0,\hspace{0.17em}1\right)$ residuals of the regression.

### Relationship between reproductive number and temperature

**Figure 1:**
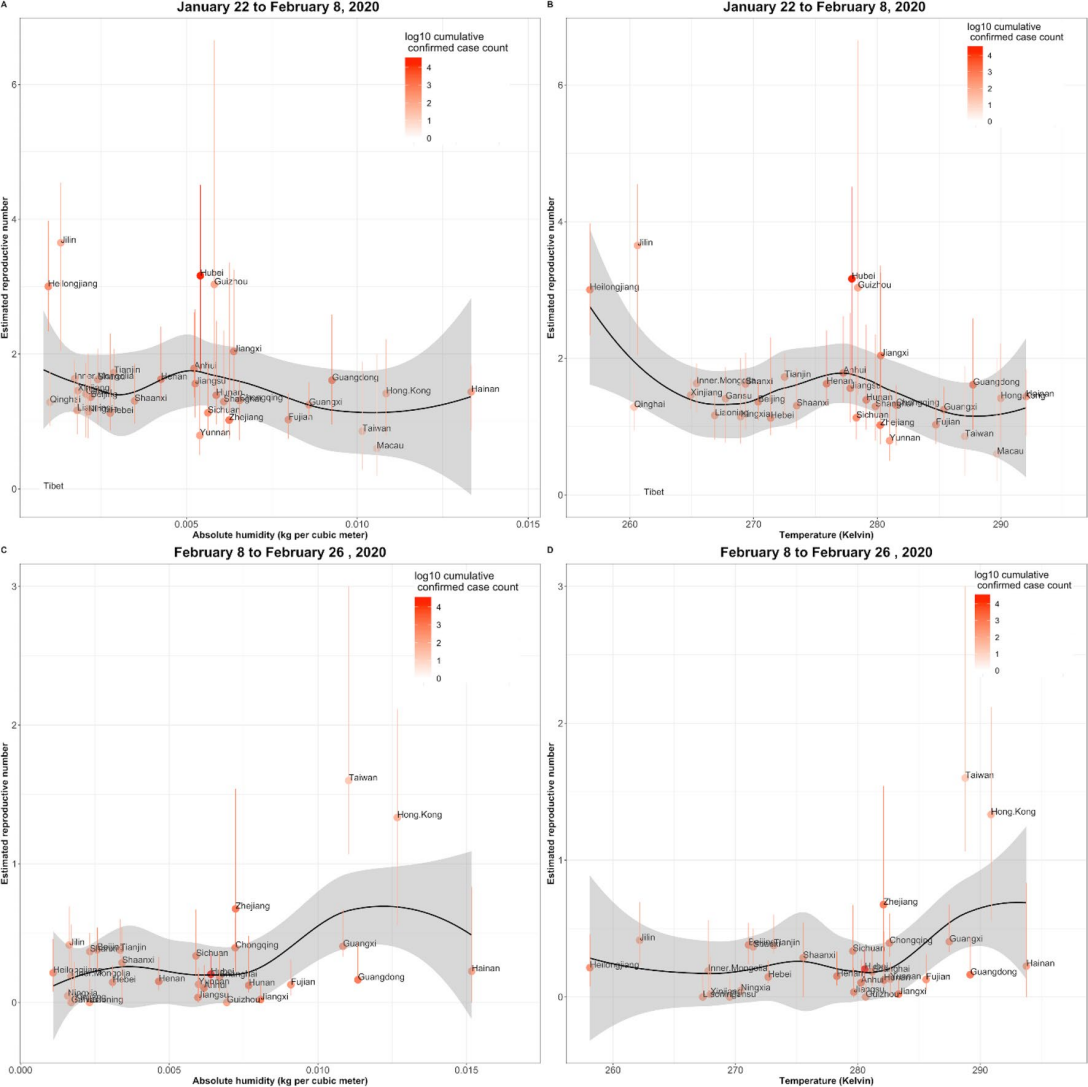
Visualization of the relationship between COVID-19 transmission as captured by R_proxy_ and temperature and humidity. The data points on the scatter plot represent the value of Rproxy (with its associated 87% confidence intervals displayed as vertical lines, obtained from the collection of *R*_*proxy*_ calculated in subsequent time windows of length *d* for each location) as a function of temperature and humidity. The black line corresponds to a Loess regression aimed at capturing the relationship between Rproxyand temperature and humidity. In addition, the color intensity (orange) of each data point shows the size of the outbreak in each location, as captured by the log of cumulative case counts.

We used a Loess regression to visually represent the relationship between the reproductive number for each province and temperature (Fig. 1). To identify the statistical relevance of this relationship we implemented a linear model using the log of the local reproductive number *R*_*proxy*_ as our response variable, and temperature as predictor and log transformation was employed to improve gaussianity (Supplementary Figure S1). The linear model was computed for both time periods described above:

$$\mathrm{log}\left({\mathrm{R}}_{\mathrm{proxy}}\left(\mathrm{j}\right)\right)={\beta {^{\prime}}}_{0}+{\beta }_{2}{\mathrm{X}}_{\mathrm{temperature}}\left(\mathrm{j}\right)+\mathrm{\epsilon {^{\prime}}}\left(\mathrm{j}\right)$$

Depending on the time period explained, *R*_*proxy*_ corresponds to $\mathrm{R}{0}_{\uptau_1}$ or $\mathrm{R}{0}_{\uptau_2}$ for the province and the city-level; ${\mathrm{X}}_{\mathrm{temperature}}$ corresponds to the temperature for the first and second time periods.

### Relationship between reproductive number and absolute humidity

As for temperature, we conducted the same analysis for absolute humidity. The linear model was:

$$\mathrm{log}\left({\mathrm{R}}_{\mathrm{proxy}}\left(\mathrm{j}\right)\right)={\beta {^{\prime}}{^{\prime}}}_{0}+{\beta }_{3}{\mathrm{X}}_{\mathrm{abs humidity}}\left(\mathrm{j}\right)+\mathrm{\epsilon {^{\prime}}}{^{\prime}}\left(\mathrm{j}\right)$$

where ${\mathrm{X}}_{\mathrm{abs humidity}}$ corresponds to the absolute humidity for the first and second time periods.

## Results

### Reproductive number proxy

In both time periods, $\uptau_1$ and $\uptau_2$, our estimates of *R*_*proxy*_ for each province within China, appeared to be consistent across the range of serial intervals we analyzed (Fig. 1). In the first time-period, most regions have a *R*_*proxy*_ estimate well above 1, signaling sustained disease transmission. *R*_*proxy*_ estimates across provinces decreased dramatically on the second time-period, many below 1, likely as a response to the multiple (non-pharmaceutical) interventions implemented by Chinese authorities.

### Data analysis (filtering) 

In the first step of our analysis, the provinces of Tibet, Qinghai and Macau were removed due to the low number of reported COVID-19 cases there. Low number of cases (and multiple zeros) led to invalid calculations (NaN) of *R*_*proxy*_. In the second step, we removed 3 provinces given that no mobility data were available: Tibet, Hong Kong and Inner Mongolia. Finally, 5 provinces were removed: Guizhou, Hubei, Heilongjiang, Jilin and Shandong given the unrealistically high value of their *R*_*proxy*_ (3.92, 3.19, 3.32, 3.57, and 4.45 respectively). At city level, 175 cities were removed due to the low number of cases (first filter) and 23 cities were removed because of the high value of their *R*_*proxy*_ (third filter). Finally, the values of *R*_*proxy*_ for countries are shown for reference: Iran ($\mathrm{R}{0}_{\uptau_1}=0$ and $\mathrm{R}{0}_{\uptau_2}=34.00$), Italy ($\mathrm{R}{0}_{\uptau_1}=0$ and $\mathrm{R}{0}_{\uptau_2}=107.2$), Singapore ($\mathrm{R}{0}_{\uptau_1}=1.85$ and $\mathrm{R}{0}_{\uptau_2}=0.39$), Japan ($\mathrm{R}{0}_{\uptau_1}=1.84$ and $\mathrm{R}{0}_{\uptau_2}=2.70$), and South Korea ($\mathrm{R}{0}_{\uptau_1}=3.11$ and $\mathrm{R}{0}_{\uptau_2}=196.97$).

### Relationship with mobility

Because Wuhan (provincial capital of Hubei) was the origin of the COVID-19 outbreak, and exported cases could only be calculated in the rest of the provinces, we excluded Hubei from our mobility analysis. As shown in Tables 1 and 2, identifying the influence of mobility on *R*_*proxy*_ can only be done after the third step of filtering. Human mobility (prior to Wuhan's lockdown) did not appear associated with *R*_*proxy*_ across Chinese provinces during time-period $\uptau_1$ (p value = 0.93). However, in the same time-period, once we excluded *R*_*proxy*_ values above 3 (third step of filtering), mobility was found to be associated with *R*_*proxy*_ (p value = 0.01).

**Table 1:** Relationship between reproductive number for the first time period $\mathrm{R}{0}_{\uptau_1}$, and mobility with the second step of filtering.

Variables	Number of observations: 28 F statistic: 0.009 P value (F statistic) : 0.927 R-squared: 0.000 Adjusted R-squared: − 0.040			
	Coefficient	Std error	T-Statistic	P value
Intercept	1.716	0.186	9.233	1.57 × 10^−9^
Mobility	− 0.01	0.139	− 0.092	0.927

**Table 2:** Relationship between reproductive number for the first time period $\mathrm{R}{0}_{\uptau_1}$, and mobility with the third step of filtering.

Variables	Number of observations: 23 F statistic: 7.528 P value (F statistic) : 0.012 R-squared: 0.264 Adjusted R-squared: 0.229			
	Coefficient	Std error	T-Statistic	P value
Intercept	1.351	0.073	18.473	1.82 × 10^−14^
Mobility	0.139	0.051	2.744	**0.012**

### Relationship with temperature

Figure 1 is a visualization of the relationship between COVID-19 transmission as captured by *R*_*proxy*_ and temperature and humidity. The data points on the scatter plot represent the value of *R*_*proxy*_ (with its associated confidence interval) as a function of temperature and humidity. The black line corresponds to a Loess regression aimed at capturing the relationship between *R*_*proxy*_ and temperature and humidity. Specifically, for the first time period, we can see that higher temperatures lead to lower rates of transmission. In addition, the color intensity (orange) of each data point shows the size of the outbreak in each location, as captured by the log of cumulative case counts.

**Figure 2:**
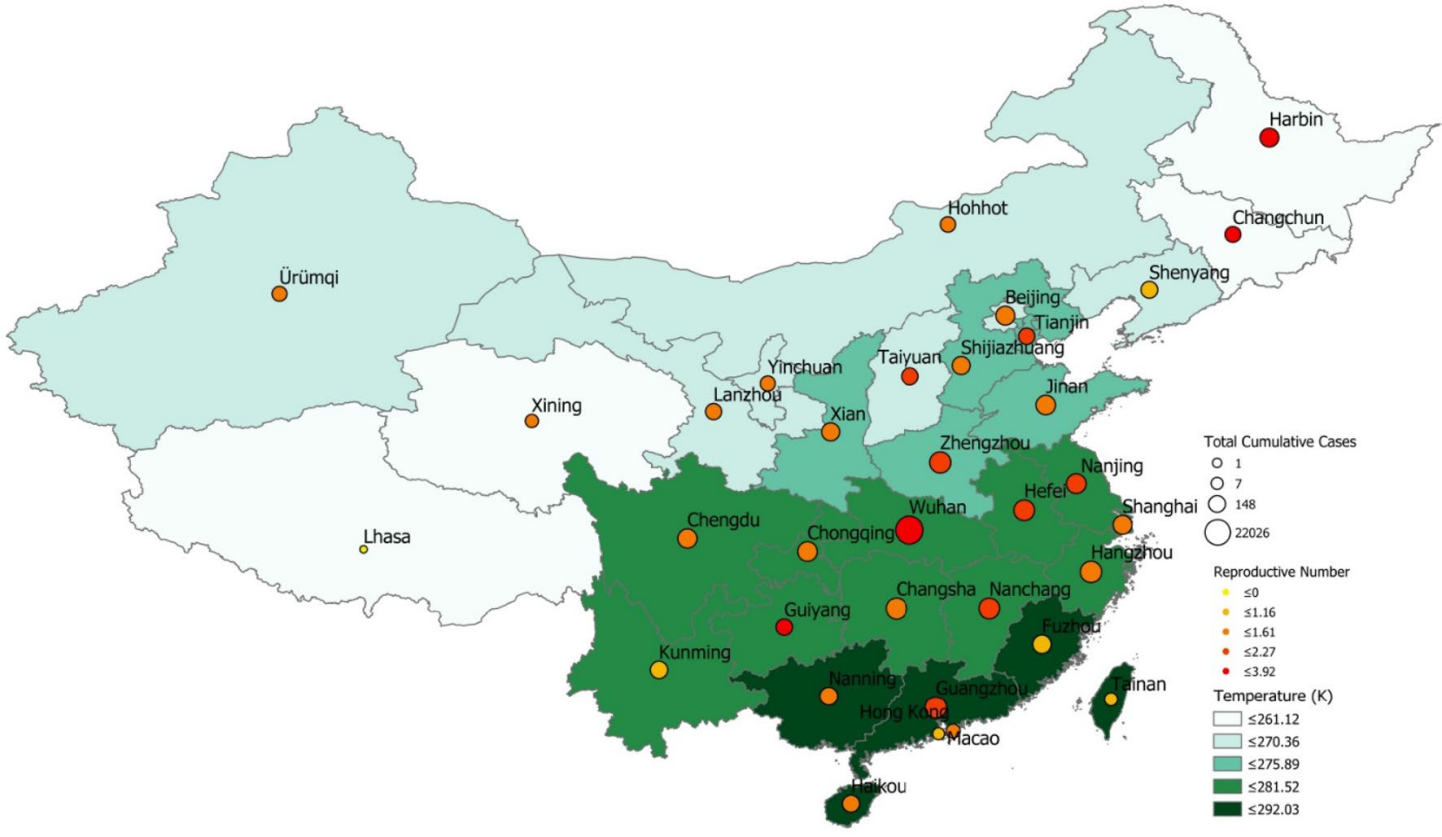
Temperature in each provincial capital vs. COVID-19 *R*_*proxy*_ estimate (calculated for the first time period). The size and color of each pin indicate cumulative cases per province and *R*_*proxy*_ range, respectively. (Map obtained with ArcMap, https://desktop.arcgis.com/en/arcmap/ version 10.2).

Regarding the results of the linear regression models, after the first step of filtering, for the time-period $\uptau_1$, temperature appeared to be associated with *R*_*proxy*_ at the 94% confidence level (Table 3). Specifically, temperature showed a negative relationship, indicating that higher temperatures appeared to have lower transmission (Fig. 2). After the two additional steps of filtering, the association between temperature and *R*_*proxy*_ became weaker or non-significant (with p values equal to 0.111 and 0.857 respectively; Tables 4 and 5). Weak to non-significant associations were observed when we conducted our analysis for the second time-period $\uptau_2$, with P values ranging from 0.118 to 0.700 (Tables 6, 7, 8). At the city-level in China the temperature appeared to be associated to *R*_*proxy*_ for the first time-period and after removing cities with low number of cases (p value = 0.01; Supplementary Table S1). After removing *R*_*proxy*_ above 3, the temperature was no longer associated with *R*_*proxy*_, with a p value equal to 0.83 (Supplementary Table S2). No associations were observed for the city-level analysis for the second time-period, with p values equal to 0.32 and 0.23 after the two steps of filtering (Supplementary Tables S3, S4).

**Table 3:** Relationship between $\mathrm{log}(\mathrm{R}{0}_{\uptau_1}$) and temperature with the first step of filtering.

Variable	Number of observations: 31 F statistic: 3.966 P value (F statistic) : 0.056 R-squared: 0.120 Adjusted R-squared: 0.090			
	Coefficient	Std error	T-Statistic	P value
Intercept	4.553	2.050	2.220	0.034
Temperature	− 0.015	0.007	− 1.991	**0.056**

**Table 4:** Relationship between $\mathrm{log}(\mathrm{R}{0}_{\uptau_1}$) and temperature with the second step of filtering.

Variables	Number of observations: 28 F statistic: 2.725 P value (F statistic) : 0.1108 R-squared: 0.095 Adjusted R-squared: 0.060			
	Coefficient	Std error	T-Statistic	P value
Intercept	4.369	2.348	1.861	0.074
Temperature	− 0.014	0.009	− 1.651	0.111

**Table 5:** Relationship between $\mathrm{log}(\mathrm{R}{0}_{\uptau_1}$) and temperature with the third step of filtering.

Variables	Number of observations: 23 F statistic: 0.033 P value (F statistic) : 0.857 R-squared: 0.002 Adjusted R-squared: − 0.046			
	Coefficient	Std error	T-Statistic	P value
Intercept	0.685	1.695	0.404	0.690
Temperature	− 0.001	0.006	− 0.183	0.857

**Table 6:** Relationship between $\mathrm{log}(\mathrm{R}{0}_{\uptau_2}$) and temperature with the first step of filtering.

Variables	Number of observations: 31 F statistic: 1.565 P value (F statistic) : 0.221 R-squared: 0.051 Adjusted R-squared: 0.018			
	Coefficient	Std Error	T-Statistic	P value
Intercept	− 10.03	6.795	− 1.476	0.151
Temperature	0.031	0.024	1.251	0.221

**Table 7:** Relationship between $\mathrm{log}(\mathrm{R}{0}_{\uptau_2}$) and temperature with the second step of filtering.

Variables	Number of observations: 28 F statistic: 0.152 P value (F statistic) : 0.700 R-squared: 0.006 Adjusted R-squared: − 0.032			
	Coefficient	Std error	T-Statistic	P value
Intercept	− 4.478	7.199	− 0.622	0.539
Temperature	0.010	0.026	0.389	0.700

**Table 8:** Relationship between $\mathrm{log}(\mathrm{R}{0}_{\uptau_2}$) and temperature with the third step of filtering.

Variables	Number of observations: 23 F statistic: 2.659 P value (F statistic) : 0.118 R-squared: 0.112 Adjusted R-squared: 0.070			
	Coefficient	Std error	T-Statistic	P value
Intercept	− 13.12	6.96	− 1.886	0.073
Temperature	0.041	0.025	1.631	0.118

### Relationship with absolute humidity

**Figure 3:**
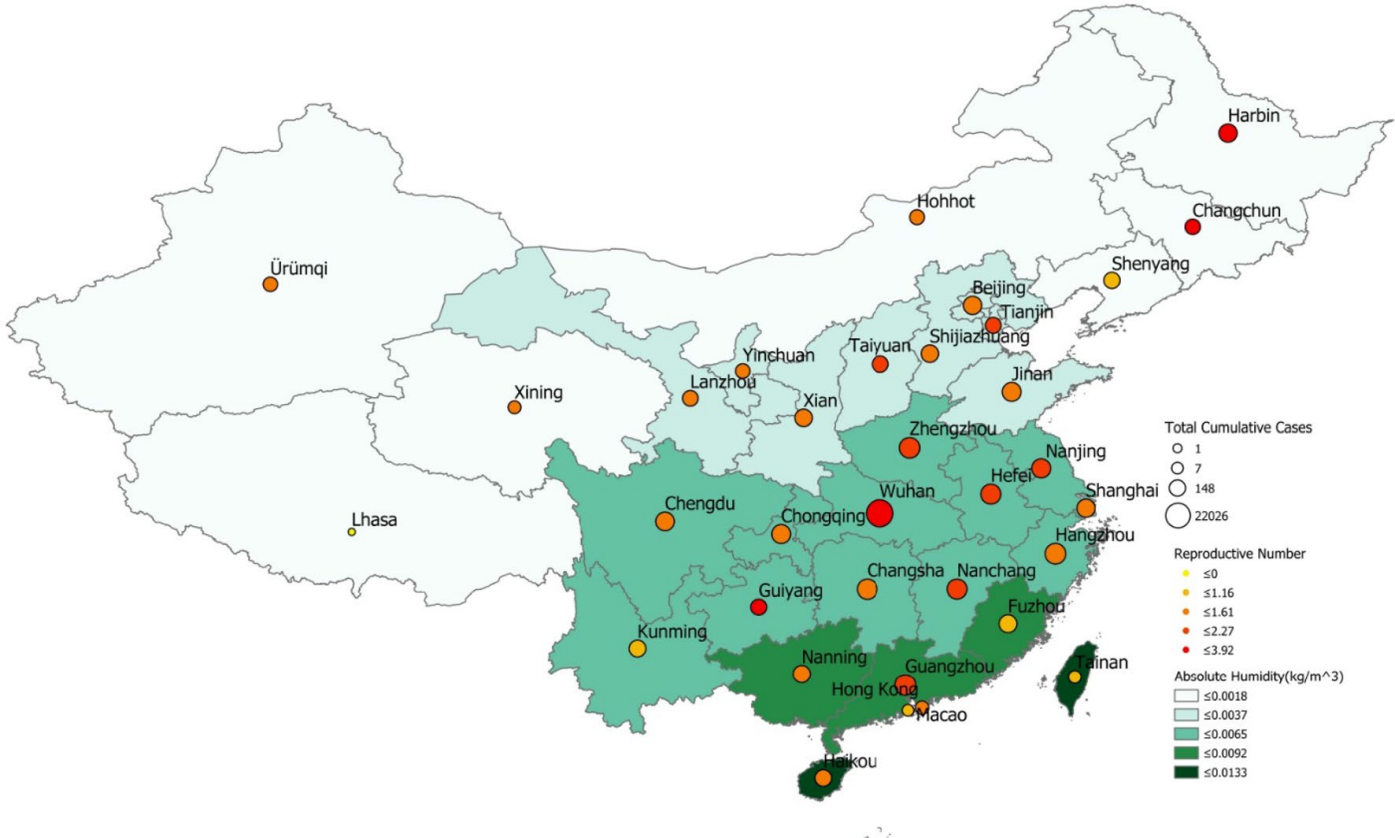
Absolute humidity in each provincial capital vs. *R*_*proxy*_ estimate (calculated for the first time period). The size and color of each pin indicate cumulative cases per province and *R*_*proxy*_ range, respectively. (Map obtained with ArcMap, https://desktop.arcgis.com/en/arcmap/ version 10.2).

In all steps of filtering at the province-level, and for both time periods, $\uptau_1$ and $\uptau_2$, absolute humidity was not associated to *R*_*proxy*_, with P values ranging between 0.161 and 0.922 (Tables 9, 10, 11, 12, 13, 14, 15). This can also be observed in Fig. 1, where the black curve (corresponding to the Loess regression) is relatively flat. Meanwhile, Fig. 3 allows us to visualize the values of *R*_*proxy*_ and humidity across regions. For cities, for time-period $\uptau_1$, and after the first step of filtering, absolute humidity appeared to be associate with *R*_*proxy*_ with a p value equal to 0.004 (Supplementary Table S5). Specifically, absolute humidity showed a negative relationship, indicating that locations with higher absolute humidity experienced lower transmission. Nevertheless, after the third step of filtering, absolute humidity was not found to be associated with *R*_*proxy*_, with a p value equal to 0.64 (Table S6). For the second time period $\uptau_2$, no associations were found either, with p values equal to 0.95 and 0.87 after the two steps of filtering, respectively (Tables S7, S8).

**Table 9:** Relationship between $\mathrm{log}(\mathrm{R}{0}_{\uptau_1}$) and absolute humidity with the first step of filtering.

Variables	Number of observations: 31 F statistic: 1.861 P value (F statistic) : 0.183 R-squared: 0.060 Adjusted R-squared: 0.028			
	Coefficient	Std Error	T-Statistic	P value
Intercept	0.618	0.125	4.945	2.95 × 10^–5^
Absolute humidity	− 28.84	21.14	− 1.364	0.183

**Table 10:** Relationship $\mathrm{log}(\mathrm{R}{0}_{\uptau_1}$) and absolute humidity with the second step of filtering.

Variables	Number of observations: 28 F statistic: 0.784 P value (F statistic) : 0.384 R-squared: 0.029 Adjusted R-squared: − 0.008			
	Coefficient	Std error	T-Statistic	P value
Intercept	0.601	0.139	4.314	2.1 × 10^–4^
Absolute humidity	− 22.25	25.132	− 0.885	0.384

**Table 11:** Relationship between $\mathrm{log}(\mathrm{R}{0}_{\uptau_1}$), and absolute humidity with the third step of filtering.

Variables	Number of observations: 23 F statistic: 0.010 P value (F statistic) : 0.922 R-squared: 0.000 Adjusted R-squared: − 0.047			
	Coefficient	Std error	T-Statistic	P value
Intercept	0.383	0.088	4.345	2.8 × 10^–4^
Absolute humidity	− 1.501	15.130	− 0.099	0.922

**Table 12:** Relationship between $\mathrm{log}(\mathrm{R}{0}_{\uptau_2}$) and absolute humidity with the first step of filtering.

Variables	Number of observations: 31 F statistic: 2.072 P value (F statistic) : 0.161 R-squared: 0.067 Adjusted R-squared: 0.035			
	Coefficient	Std error	T-Statistic	P value
Intercept	− 2.006	0.389	− 5.156	1.65 × 10^–5^
Absolute humidity	79.68	55.35	1.439	0.161

**Table 13:** Relationship between $\mathrm{log}(\mathrm{R}{0}_{\uptau_2}$) and absolute humidity with the second step of filtering.

Variables	Number of observations: 28 F statistic: 0.192 P value (F statistic) : 0.665 R-squared: 0.007 Adjusted R-squared: − 0.031			
	Coefficient	Std Error	T-Statistic	P value
Intercept	− 1.827	0.404	− 4.520	1.19 × 10^–4^
Absolute humidity	26.669	60.93	0.438	0.665

**Table 14:** Relationship between $\mathrm{log}(\mathrm{R}{0}_{\uptau_2}$), and absolute humidity with the third step of filtering.

Variables	Number of observations: 23 F statistic: 1.939 P value (F statistic) : 0.178 R-squared: 0.085 Adjusted R-squared: 0.041			
	Coefficient	Std error	T-Statistic	P value
Intercept	− 2.20	0.355	− 6.211	3.67 × 10^–6^
Absolute humidity	70.98	50.97	1.393	0.178

**Table 15:** Summary of the principal results (P value, R^2^) of the linear regressions.

Model	Time period	Filtering	P value	R squared
Mobility	τ_1_	2nd step	0.927	0.000
Mobility	τ_1_	3rd step	**0.012**	0.264
Temperature	τ_1_	1st step	**0.056**	0.120
Temperature	τ_1_	2nd step	0.111	0.095
Temperature	τ_1_	3rd step	0.857	0.002
Temperature	τ_2_	1st step	0.221	0.051
Temperature	τ_2_	2nd step	0.700	0.006
Temperature	τ_2_	3rd step	0.118	0.112
Absolute humidity	τ_1_	1st step	0.183	0.060
Absolute humidity	τ_1_	2nd step	0.384	0.029
Absolute humidity	τ_1_	3rd step	0.922	0.000
Absolute humidity	τ_2_	1st step	0.161	0.067
Absolute humidity	τ_2_	2nd step	0.665	0.007
Absolute humidity	τ_2_	3rd step	0.178	0.085

The numbers in bold correspond to p-values less than or about 0.05.

## Discussion

Ambient temperature appears to be associated to COVID-19 transmission (as captured by our proxy of R) during the first time-period (January 22, 2020–February 8, 2020) in both spatial resolutions and in the absence of any data filtering. Specifically, temperature showed a negative relationship, indicating that higher temperatures appeared to have lower COVID-19 transmission. These results were not robust to filtering techniques aimed at removing noisy values such as unrealistically high values of *R*_*proxy*_ (more than 3). In an effort to identify if transmission rates could be explained by the rate of case importations at the province-level, we analyzed if mobility from Wuhan to each province could explain the spatial variability of *R*_*proxy*_ during the first time-period. Our results showed no associations between mobility and *R*_*proxy*_ in the absence of data filtering but showed that *R*_*proxy*_ could be explained by mobility when removing values of *R*_*proxy*_ larger than 3. Finally, our analysis suggests that absolute humidity was not robustly associated with *R*_*proxy*_, but these results need to be interpreted carefully given the monotonic functional relationship between humidity and temperature (Clausius–Clapeyron relation). In other words, if temperature were associated to COVID-19 transmission, very likely absolute humidity would play a role.

### Limitations

Our estimates of the observed *R*_*proxy*_ across locations were calculated using available and likely incomplete reported case count data, with date of reporting, rather than date of onset, which adds noise to the estimation. In addition, the relatively short time length of the current outbreak, combined with imperfect daily reporting practices, make our results vulnerable to changes as more data becomes available. We have assumed that travel limitations and other containment interventions have been implemented consistently across provinces and have had similar impacts (thus population mixing and contact rates are assumed to be comparable), and have ignored the fact that different places may have different reporting practices. Further improvements could incorporate data augmentation techniques that may be able to produce historical time series with likely estimates of case counts based on onset of disease rather than reporting dates. This, along with more detailed estimates of the serial interval distribution, could yield more realistic estimates of *R*. In addition, while the low R^2^ values from our models show that each individual variable is not enough to explain the variability of COVID-19 transmission rate, we considered that finding statistically significant relationships could help us achieve our goal. In fact, if the goal were to design a model to explain the variance of Rt one would likely require more input variables, for example the density of population in each area, people’s behaviour (regarding mask-wearing adoption, for example) or socio economic factors, etc. Future studies should incorporate all these variables to further characterize transmission. Finally, further experimental work needs to be conducted to better understand the mechanisms of transmission for COVID-19. Mechanistic understanding of transmission could lead to a coherent justification of our findings.

### Conclusion

Despite the above limitations, our early and near-real-time analysis regarding the impact of environmental factors on COVID-19 transmission in China could provide useful implications for policymakers and the public worldwide. Sustained transmission and rapid growth of cases were observed over a range of temperatures and humidity conditions ranging from cold and dry provinces in China, such as Jilin and Heilongjiang, to tropical locations, such as Guangxi and Taiwan during the first time-period (τ1, from January 22 to February 8, 2020). Our results show that weather alone cannot explain, in a robust way, the variability of the reproductive number in Chinese provinces or cities. Moreover, drastic reductions in transmission were observed during the second half of February, likely due to the strict non-pharmaceutical interventions imposed across China. In addition, we can see that all these findings have been confirmed in these past few months. Further studies on the effects of environmental factors on COVID-19 will be possible as more data is collected in multiple affected geographies during this COVID-19 outbreak.

## Supplementary information

Supplementary Information

## Data Availability

All codes and data are made available via the Harvard dataverse: https://doi.org/10.7910/DVN/SRHAJN
