# Supplementary material for: The role of environmental factors on transmission rates of the COVID-19 outbreak: an initial assessment in two spatial scales
Source: Sci Rep. 2020 Oct 12;10:17002. doi: 10.1038/s41598-020-74089-7 (PMC7552413; doi:10.1038/s41598-020-74089-7)

**The role of environmental factors on transmission rates of the COVID-19 outbreak: An initial assessment in two spatial scales.**

**Canelle Poirier^1,2^, Wei Luo^1,2^, Maimuna S. Majumder^1,2^, Dianbo Liu^1,2^,**

**Kenneth D. Mandl^1,2,3^, Todd A. Mooring^4^, Mauricio Santillana^1,2,5,*^**

^1^ Computational Health Informatics Program, Boston Children's Hospital, Boston MA 02215

^2^ Department of Pediatrics, Harvard Medical School, Boston MA 02215

^3^ Department of Biomedical Informatics, Harvard Medical School, Boston MA USA 02215

^4^ Department of Earth and Planetary Sciences, Harvard University, Cambridge MA 02138

^5^ Harvard T.H. Chan School of Public Health, Boston MA 02215

Corresponding authors: Canelle Poirier, [canelle.poirier@outlook.fr](mailto:canelle.poirier@outlook.fr) and Mauricio Santillana, [msantill@g.harvard.edu](mailto:msantill@g.harvard.edu)

**Supplementary Materials**

**Weather data.**

Near-surface water vapor density is not part of the standard ERA5 output. Instead, it must be computed using the data that are available and knowledge of atmospheric physics. The relevant available data are 2-m air temperature (T_2_, i.e. temperature at a nominal height of 2 m above the ground), 2-m dew point temperature (T_d_), and surface pressure (p_s_).

Together, T_2_ and T_d_ encode the near-surface (water) vapor pressure e_2_. Using the definition of dew point (21), we calculated $e_{2}=e_{s}\left( T_{d} \right)$

where $e_{s}\left( T \right)$ is the saturation vapor pressure (with respect to liquid water) as a function of temperature. The exact form of this function is taken from the documentation of the numerical model underlying ERA5 (22)

:

$$e_{s}\left( T \right)=611.21\exp\left( \frac{17.502\left( T-273.16 \right)}{T-32.19} \right)$$

where T has units of kelvin and e_s_ has units of pascals. Note that occasionally a given space-time point in the ERA5 data will have T_d_>T_2_. This is purely a numerical artifact---the atmosphere is not really supersaturated (Kevin Marsh, pers. comm., 26 February 2020). When this occurs we reduce T_d_ to T_2_ (i.e., reduce relative humidity to 100%) before proceeding with further analysis. Near-surface water vapor density $\rho_{v2}$may then be computed using the ideal gas law (21):

$$\rho_{v2}=\frac{e_{2}}{R_{v}T_{2}}$$

where the value of the water vapor gas constant R_v_=461.525 J/kg/K is taken from (22). Time-averaged absolute humidity (and temperature) data are desired for analysis. The above thermodynamic equations are clearly nonlinear, so the time averaging operation does not commute with the conversion from T_d_ to $\rho_{v2}.$We thus computed $\rho_{v2}$for every single hour in the time intervals of interest, then took time averages as needed.

**Appendix Tables.**

Table S1. Relationship between $log(R0_{\tau1}$) and temperature with the first step of filtering (City level).

Number of observations 170

F-statistic 6.454

P-value (F-statistic) 0.012

R-squared 0.037

Adjusted R-squared 0.031

| Variable | Coefficient | Std Error | T-Statistic | P-value |
| --- | --- | --- | --- | --- |
| Intercept | 5.524 | 2.015 | 2.741 | 0.007 |
| Temperature | -0.018 | 0.007 | -2.540 | **0.012** |

Table S2. Relationship between $log(R0_{\tau1}$) and temperature with the third step of filtering. (City level)

Number of observations 147

F-statistic 0.049

P-value (F-statistic) 0.825

R-squared 0.000

Adjusted R-squared -0.007

| Variable | Coefficient | Std Error | T-Statistic | P-value |
| --- | --- | --- | --- | --- |
| Intercept | -0.169 | 1.758 | -0.096 | 0.923 |
| Temperature | 0.0014 | 0.006 | 0.221 | 0.825 |

Table S3. Relationship between $log(R0_{\tau2}$) and temperature with the first step of filtering. (City level)

Number of observations 170

F-statistic 0.996

P-value (F-statistic) 0.320

R-squared 0.006

Adjusted R-squared 0.000

| Variable | Coefficient | Std Error | T-Statistic | P-value |
| --- | --- | --- | --- | --- |
| Intercept | 1.731 | 3.199 | 0.541 | 0.589 |
| Temperature | -0.011 | 0.011 | -0.998 | 0.320 |

Table S4. Relationship between $log(R0_{\tau2}$) and temperature with the third step of filtering. (City level)

Number of observations 147

F-statistic 1.450

P-value (F-statistic) 0.230

R-squared 0.010

Adjusted R-squared 0.003

| Variable | Coefficient | Std Error | T-Statistic | P-value |
| --- | --- | --- | --- | --- |
| Intercept | 2.630 | 3.394 | 0.775 | 0.440 |
| Temperature | -0.015 | 0.012 | -1.204 | 0.230 |

Table S5. Relationship between $log(R0_{\tau1}$) and absolute humidity with the first step of filtering. (City level)

Number of observations 170

F-statistic 8.658

P-value (F-statistic) 0.004

R-squared 0.049

Adjusted R-squared 0.043

| Variable | Coefficient | Std Error | T-Statistic | P-value |
| --- | --- | --- | --- | --- |
| Intercept | 3.077 | 0.400 | 7.699 | 1.12*10^-12^ |
| Absolute Humidity | -201.7 | 68.55 | -2.942 | **0.004** |

Table S6. Relationship $log(R0_{\tau1}$) and absolute humidity with the third step of filtering. (City level)

Number of observations 147

F-statistic 0.225

P-value (F-statistic) 0.636

R-squared 0.002

Adjusted R-squared -0.005

| Variable | Coefficient | Std Error | T-Statistic | P-value |
| --- | --- | --- | --- | --- |
| Intercept | 1.345 | 0.121 | 11.086 | 2.0*10^-16^ |
| Absolute Humidity | 9.748 | 20.571 | 0.474 | 0.636 |

Table S7. Relationship between $log(R0_{\tau2}$) and absolute humidity with the first step of filtering. (City level)

Number of observations 170

F-statistic 0.004

P-value (F-statistic) 0.947

R-squared 0.000

Adjusted R-squared -0.006

| Variable | Coefficient | Std Error | T-Statistic | P-value |
| --- | --- | --- | --- | --- |
| Intercept | 0.477 | 0.200 | 2.390 | 0.018 |
| Absolute Humidity | -1.875 | 28.092 | -0.067 | 0.947 |

Table S8. Relationship between $log(R0_{\tau2}$) and absolute humidity with the third step of filtering. (City level)

Number of observations 147

F-statistic 0.026

P-value (F-statistic) 0.872

R-squared 0.000

Adjusted R-squared -0.007

| Variable | Coefficient | Std Error | T-Statistic | P-value |
| --- | --- | --- | --- | --- |
| Intercept | 0.356 | 0.065 | 5.509 | 1.6*10^-7^ |
| Absolute Humidity | 1.45 | 8.983 | 0.161 | 0.872 |

**Appendix Figures.**

Figure S1. Histogram of residuals for the linear regressions with and without log transformation of the predictors (Mobility and Temperature)


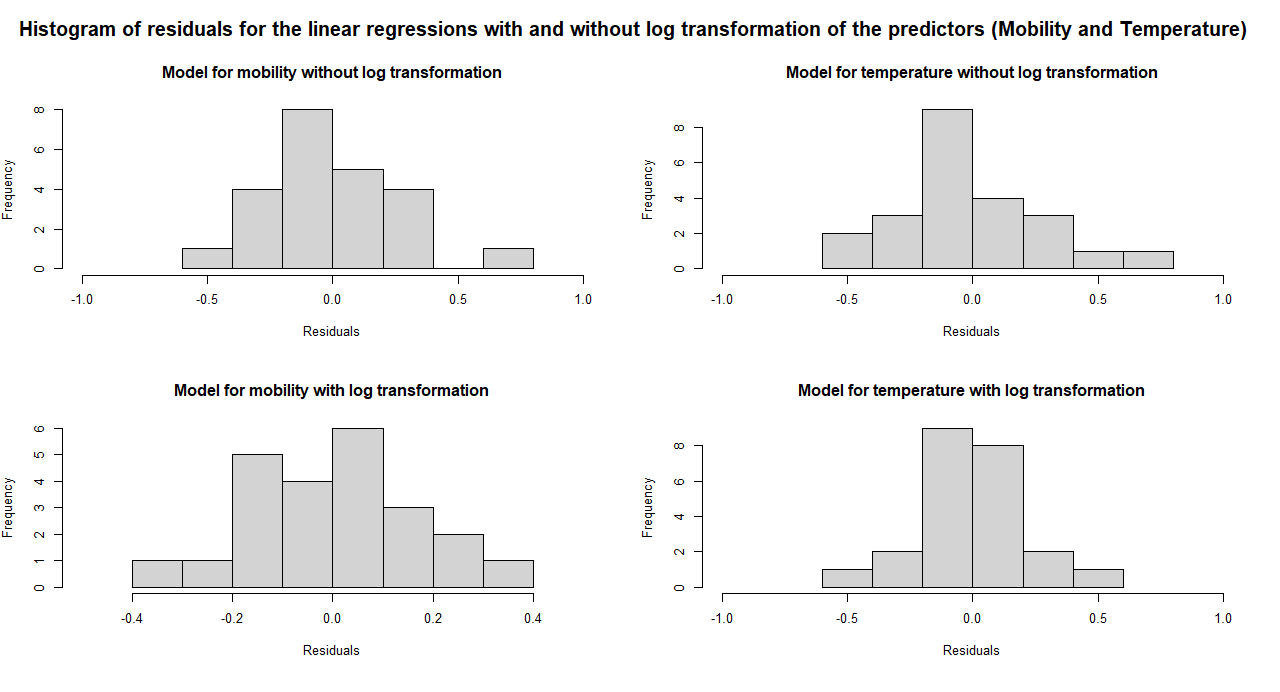


Figure S2. Temperature in each provincial capital vs. COVID-19 *R_proxy_* estimate (calculated for the second time period). The size and color of each pin indicate cumulative cases per province and *R_proxy_* range, respectively. (Map obtained with ArcMap, <https://desktop.arcgis.com/en/arcmap/> version 10.2)


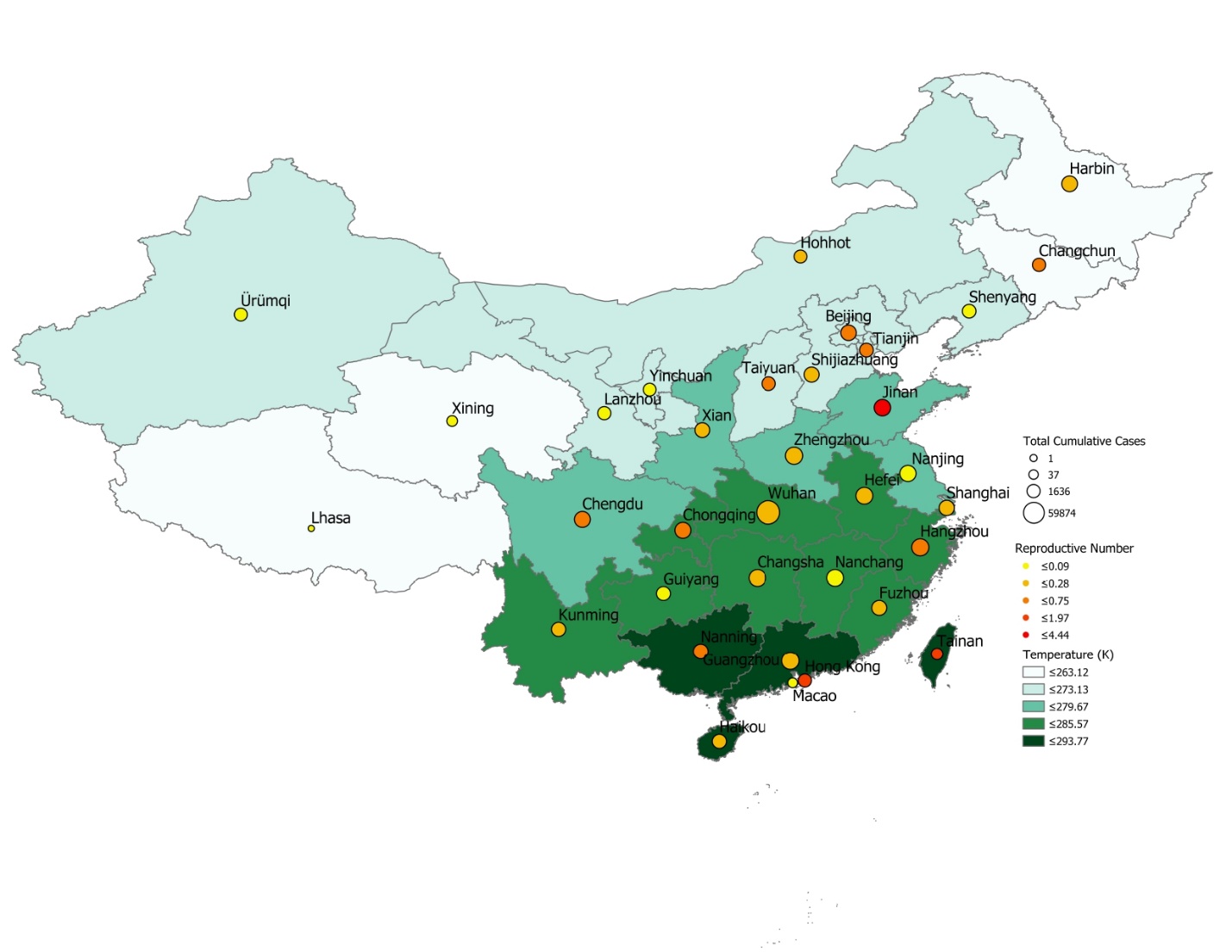


Figure S3. Absolute humidity in each provincial capital vs. COVID-19 *R_proxy_* estimate (calculated for the second time period). (Map obtained with ArcMap, <https://desktop.arcgis.com/en/arcmap/> version 10.2)


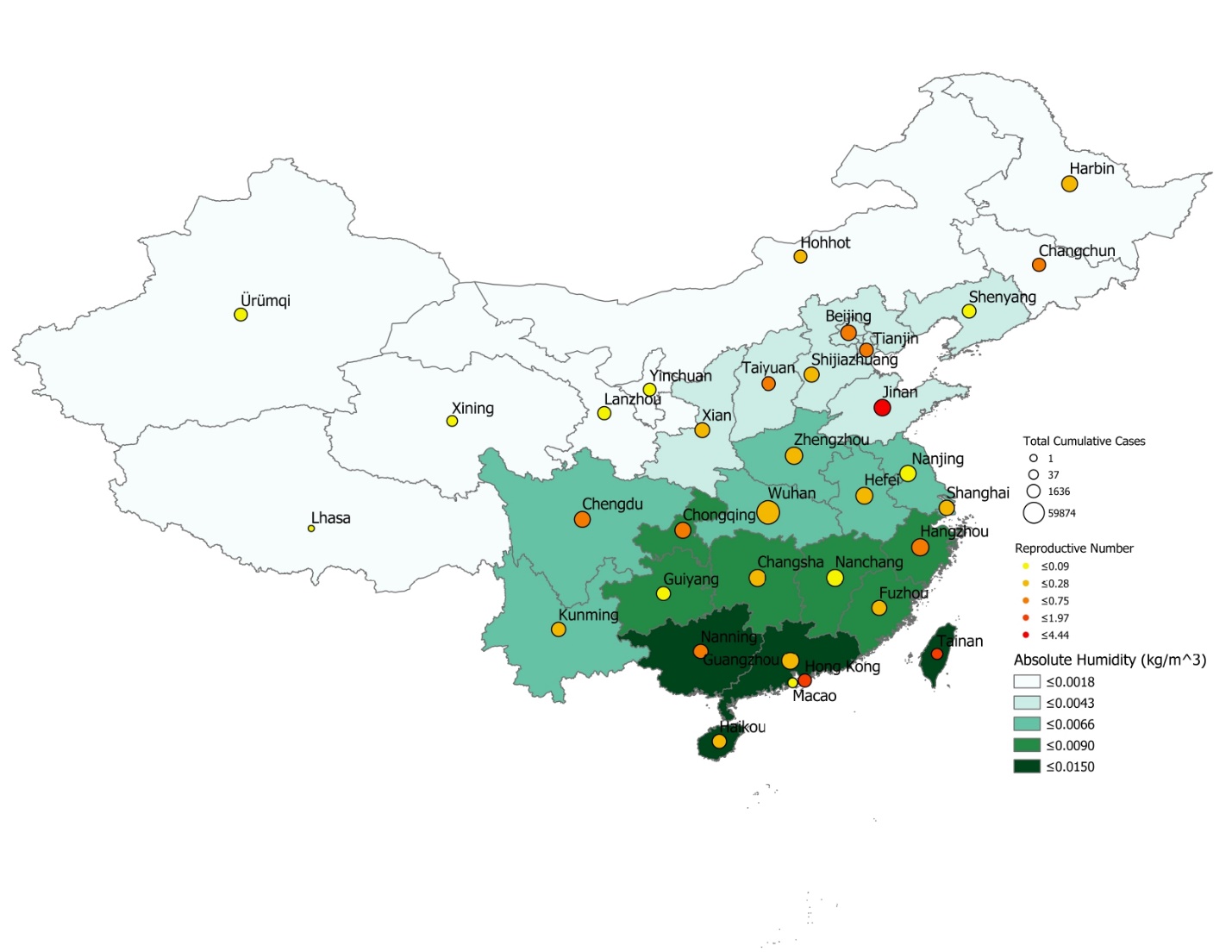

Supplement: Supplementary file 1 — Supplementary Information [file 41598_2020_74089_MOESM1_ESM.docx]
